# Supplementary figures and images for: A trial-based cost-utility analysis of sugemalimab vs. placebo as consolidation therapy for unresectable stage III NSCLC in China
Source: PLoS One. 2023 Jun 1;18(6):e0286595. doi: 10.1371/journal.pone.0286595 (PMC10234548; doi:10.1371/journal.pone.0286595)

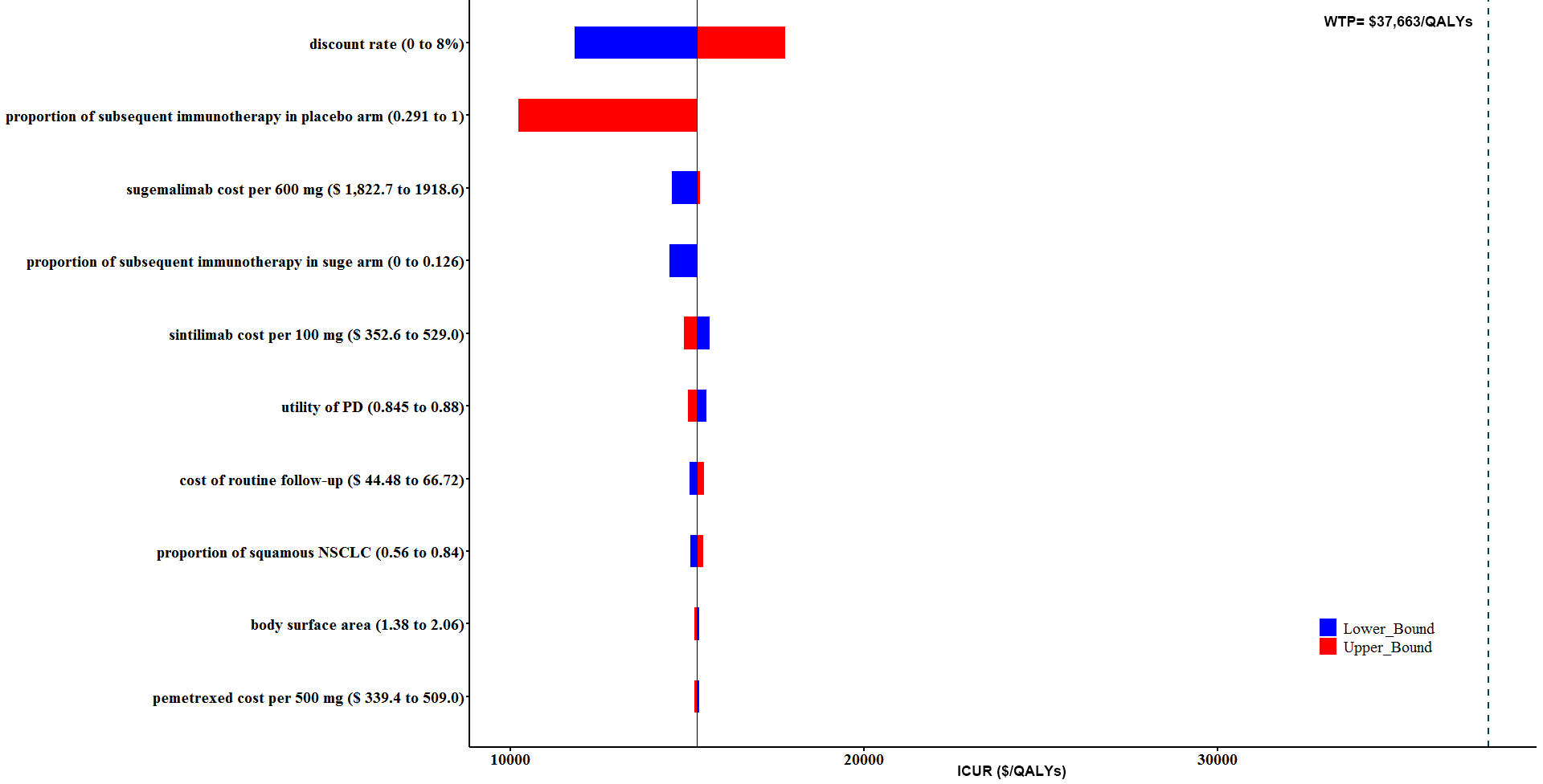

Supplement: S1 Fig — cCRT, concurrent chemoradiotherapy; PAP: Patient assistance program. (TIF) [file pone.0286595.s001.tif]

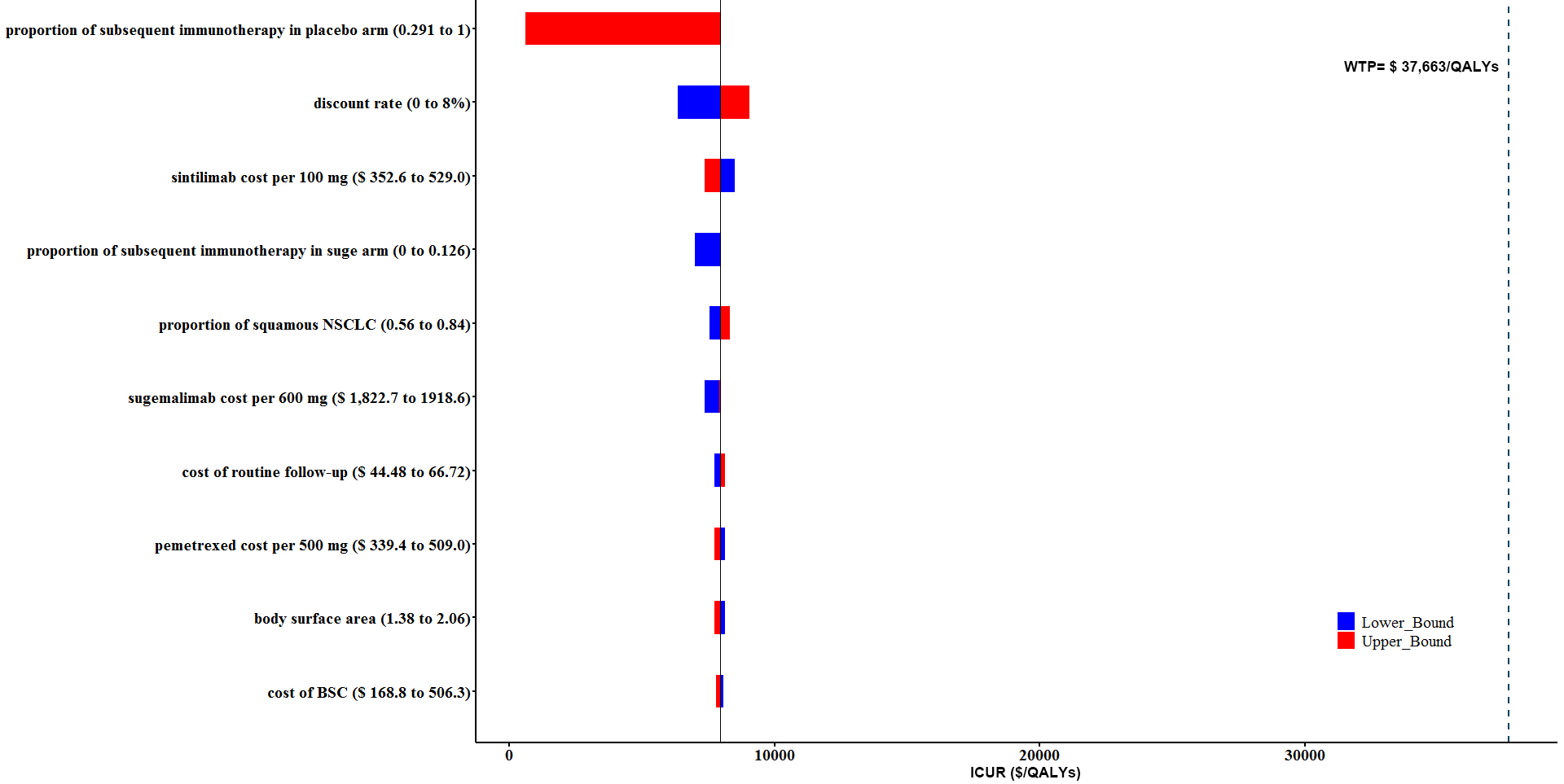

Supplement: S2 Fig — sCRT, sequential chemoradiotherapy; PAP: Patient assistance program. (TIF) [file pone.0286595.s002.tif]
