# Supplementary material for: A trial-based cost-utility analysis of sugemalimab vs. placebo as consolidation therapy for unresectable stage III NSCLC in China
Source: PLoS One. 2023 Jun 1;18(6):e0286595. doi: 10.1371/journal.pone.0286595 (PMC10234548; doi:10.1371/journal.pone.0286595)
Supplement: S1 Table — *, best fitted model; cCRT, concurrent chemoradiotherapy; sCRT, sequential chemoradiotherapy; Suge: Sugemalimab; PC; AIC, Akaike information criterion; BIC, Bayesian information criterion; PFS, progression-free survival; OS, overall survival; Suge, sugemalimab. (DOCX) [file pone.0286595.s003.docx]

| **S1 Table. AIC and BIC of parametric distributions for cCRT and sCRT curves.** | | | | | |
| --- | --- | --- | --- | --- | --- |
|  | Suge | |  | Placebo | |
| subgroup | AIC | BIC |  | AIC | BIC |
| **cCRT, OS** | | | | | |
| Exponential | 592 | 595 |  | 344 | 347 |
| Weibull | 586 | 592 |  | 335 | 340 |
| Gompertz | 590 | 596 |  | 337 | 341 |
| Log-logistic | 585 | 591 |  | 336 | 341 |
| Log-normal | 589 | 595 |  | 345^*^ | 350^*^ |
| MCM- Exponential | 594 | 600 |  | 346 | 351 |
| MCM-Weibull | 586 | 595 |  | 337 | 345 |
| MCM-Gompertz | 586 | 595 |  | 338 | 346 |
| MCM-Log-logistic | 586 | 596 |  | 338 | 345 |
| MCM-Log-normal | 590 | 600 |  | 347 | 354 |
| MCM-Gamma | 587^*^ | 596^*^ |  | 338 | 346 |
| MCM- Generalised gamma | 587 | 600 |  | 339 | 349 |
| **cCRT, PFS** | | | | | |
| Exponential | 799 | 802 |  | 406 | 409 |
| Weibull | 800 | 806 |  | 408 | 413 |
| Gompertz | 792^*^ | 798^*^ |  | 405^*^ | 410^*^ |
| Log-logistic | 791 | 797 |  | 398 | 403 |
| Log-normal | 783 | 790 |  | 395 | 399 |
| MCM- Exponential | 792 | 798 |  | 403 | 408 |
| MCM-Weibull | 791 | 801 |  | 392 | 399 |
| MCM-Gompertz | - | - |  | 401 | 408 |
| MCM-Log-logistic | 785 | 794 |  | 385 | 393 |
| MCM-Log-normal | 779 | 788 |  | 383 | 391 |
| MCM-Gamma | 789 | 799 |  | 388 | 395 |
| MCM- Generalised gamma | 769 | 782 |  | - | - |
| **sCRT, OS** | | | | | |
| Exponential | 338 | 340 |  | 215 | 217 |
| Weibull | 339 | 344 |  | 211 | 215 |
| Gompertz | 338 | 343 |  | 213 | 217 |
| Log-logistic | 340 | 345 |  | 211^*^ | 214^*^ |
| Log-normal | 342 | 347 |  | 211 | 215 |
| MCM- Exponential | 340 | 344 |  | 217 | 220 |
| MCM-Weibull | 341 | 348 |  | 212 | 217 |
| MCM-Gompertz | 340 | 347 |  | 212 | 217 |
| MCM-Log-logistic | 342 | 349 |  | 213 | 218 |
| MCM-Log-normal | 344 | 352 |  | 213 | 218 |
| MCM-Gamma | 341^*^ | 348^*^ |  | 213 | 218 |
| MCM- Generalised gamma | 339 | 349 |  | 212 | 218 |
| **sCRT, PFS** | | | | | |
| Exponential | 506 | 509 |  | 240 | 242 |
| Weibull | 503 | 507 |  | 242 | 245 |
| Gompertz | 494^*^ | 499^*^ |  | 237 | 240 |
| Log-logistic | 492 | 496 |  | 225 | 229 |
| Log-normal | 487 | 492 |  | 226 | 230 |
| MCM- Exponential | 497 | 502 |  | 233 | 237 |
| MCM-Weibull | 499 | 506 |  | 225 | 230 |
| MCM-Gompertz | 496 | 503 |  | 231 | 236 |
| MCM-Log-logistic | 489 | 496 |  | 220 | 225 |
| MCM-Log-normal | 485 | 492 |  | 217 | 222 |
| MCM-Gamma | 499 | 506 |  | 222 | 227 |
| MCM- Generalised gamma | 479 | 489 |  | 211^*^ | 218^*^ |
| *, best fitted model; cCRT, concurrent chemoradiotherapy; sCRT, sequential chemoradiotherapy; Suge: sugemalimab; PC; AIC, Akaike information criterion; BIC, Bayesian information criterion; PFS, progression-free survival; OS, overall survival; Suge, sugemalimab. | | | | | |
