# Supplementary material for: A trial-based cost-utility analysis of sugemalimab vs. placebo as consolidation therapy for unresectable stage III NSCLC in China
Source: PLoS One. 2023 Jun 1;18(6):e0286595. doi: 10.1371/journal.pone.0286595 (PMC10234548; doi:10.1371/journal.pone.0286595)
Supplement: Attachment — Submitted filename: PONE-D-23-01774_reviewer.pdf [file pone.0286595.s005.pdf]

PLOS ONE

A trial-based cost-effectiveness analysis of sugemalimab vs. placebo as consolidation therapy for unresectable stage III NSCLC in China

--Manuscript Draft--

**Manuscript Number:**	PONE-D-23-01774
**Article Type:**	Research Article
**Full Title:**	A trial-based cost-effectiveness analysis of sugemalimab vs. placebo as consolidation therapy for unresectable stage III NSCLC in China
**Short Title:**	Cost-effectiveness of sugemalimab for NSCLC
**Corresponding Author:**	Wei Li<br>Huazhong University of Science and Technology<br>Wuhan, CHINA
**Keywords:**	sugemalimab; cost-effectiveness; NSCLC; GEMSTONE-301; consolidation therapy
**Abstract:**	Objective:<br>To assess the cost-effectiveness of sugemalimab vs. placebo in post-chemoradiotherapy patients with locally advanced, unresectable stage III NSCLC from the perspective of the Chinese healthcare system.<br>Methods:<br>A 3-state Markov model with a 3-week cycle length was performed to appraise the incremental cost-effectiveness ratio (ICER) of sugemalimab consolidation therapy based on the GEMSTONE-301 clinical trial (data cutoff March 1, 2022) over a 21-year time horizon. Costs and health utilities were obtained from local databases and published articles. Sensitivity and scenario analyses were adopted to evaluate the model uncertainty. Internal and external data sources were used to justify the plausibility of the extrapolated portion of the survival model chosen.<br>Results:<br>In comparison with the placebo, sugemalimab consolidation therapy was not cost-effective as it yielded an ICER value of $91,072 and $56,956 for the concurrent chemoradiotherapy (cCRT) and the sequential chemoradiotherapy (sCRT) population at the willingness-to-pay (WTP) threshold of $37,663/QALYs, respectively. When taking the sugemalimab patient assistance program (PAP) into consideration, sugemalimab consolidation therapy was cost-effective with an ICER dramatic decreases below the WTP. Sensitivity analysis demonstrated that the ICER was most sensitive to the discount rate and subsequent treatment. However, none of the sensitive parameters could affect the cost-effective conclusions without or with PAP. Scenario analysis revealed that the model was particularly affected by assumptions regarding discount in sugemalimab, choice of parametric survival models, mean duration and choice of subsequent immunotherapy.<br>Conclusions:<br>From the perspective of Chinese healthcare system, sugemalimab consolidation therapy was not a cost-effective strategy in cCRT and sCRT patients with unresectable stage III NSCLC. Given that the sugemalimab PAP was available, sugemalimab consolidation therapy became a cost-effective option.
**Order of Authors:**	Wei Li
	Li Wan
**Additional Information:**	
**Question**	**Response**
**Financial Disclosure**<br><br>Enter a financial disclosure statement that describes the sources of funding for the work included in this submission. Review the submission guidelines for detailed	This work was supported by the Maternal and Child Health Hospital of Hubei Province Research Project (No. 2021SFYM030). The funders had no role in study design, data collection and analysis, decision to publish, or preparation of the manuscript.

Powered by Editorial Manager® and ProduXion Manager® from Aries Systems Corporation

requirements. View published research articles from *PLOS ONE* for specific examples.<br><br>This statement is required for submission and **will appear in the published article** if the submission is accepted. Please make sure it is accurate.<br><br>**Unfunded studies**<br>Enter: *The author(s) received no specific funding for this work.*<br><br>**Funded studies**<br>Enter a statement with the following details:<br>• Initials of the authors who received each award<br>• Grant numbers awarded to each author<br>• The full name of each funder<br>• URL of each funder website<br>• Did the sponsors or funders play any role in the study design, data collection and analysis, decision to publish, or preparation of the manuscript?<br>• **NO** - Include this sentence at the end of your statement: *The funders had no role in study design, data collection and analysis, decision to publish, or preparation of the manuscript.*<br>• **YES** - Specify the role(s) played.<br><br>* typeset	
**Competing Interests**<br><br>Use the instructions below to enter a competing interest statement for this submission. On behalf of all authors, disclose any competing interests that could be perceived to bias this work—acknowledging all financial support and any other relevant financial or non-financial competing interests.<br><br>This statement is required for submission and **will appear in the published article** if the submission is accepted. Please make sure it is accurate and that any funding sources listed in your Funding Information later in the submission form are also declared in your Financial Disclosure	The authors have declared that no competing interests exist

statement.<br><br>View published research articles from *PLOS ONE* for specific examples.<br><br>**NO authors have competing interests**<br><br>Enter: *The authors have declared that no competing interests exist.*<br><br>**Authors with competing interests**<br><br>Enter competing interest details beginning with this statement:<br><br>*I have read the journal's policy and the authors of this manuscript have the following competing interests: [insert competing interests here]*<br><br>* typeset	
**Ethics Statement**<br><br>Enter an ethics statement for this submission. This statement is required if the study involved:<br><br>• Human participants<br>• Human specimens or tissue<br>• Vertebrate animals or cephalopods<br>• Vertebrate embryos or tissues<br>• Field research<br><br>Write "N/A" if the submission does not require an ethics statement.<br><br>General guidance is provided below. Consult the submission guidelines for detailed instructions. **Make sure that all information entered here is included in the Methods section of the manuscript.**	N/A

**Format for specific study types**<br><br>**Human Subject Research (involving human participants and/or tissue)**<br>• Give the name of the institutional review board or ethics committee that approved the study<br>• Include the approval number and/or a statement indicating approval of this research<br>• Indicate the form of consent obtained (written/oral) or the reason that consent was not obtained (e.g. the data were analyzed anonymously)<br><br>**Animal Research (involving vertebrate animals, embryos or tissues)**<br>• Provide the name of the Institutional Animal Care and Use Committee (IACUC) or other relevant ethics board that reviewed the study protocol, and indicate whether they approved this research or granted a formal waiver of ethical approval<br>• Include an approval number if one was obtained<br>• If the study involved *non-human primates*, add *additional details* about animal welfare and steps taken to ameliorate suffering<br>• If anesthesia, euthanasia, or any kind of animal sacrifice is part of the study, include briefly which substances and/or methods were applied<br><br>**Field Research**<br><br>Include the following details if this study involves the collection of plant, animal, or other materials from a natural setting:<br>• Field permit number<br>• Name of the institution or relevant body that granted permission	
**Data Availability**<br><br>Authors are required to make all data underlying the findings described fully available, without restriction, and from the time of publication. PLOS allows rare exceptions to address legal and ethical concerns. See the PLOS Data Policy and FAQ for detailed information.	Yes - all data are fully available without restriction

A Data Availability Statement describing where the data can be found is required at submission. Your answers to this question constitute the Data Availability Statement and **will be published in the article**, if accepted.<br><br>**Important:** Stating 'data available on request from the author' is not sufficient. If your data are only available upon request, select 'No' for the first question and explain your exceptional situation in the text box.<br><br>Do the authors confirm that all data underlying the findings described in their manuscript are fully available without restriction?	
**Describe where the data may be found in full sentences. If you are copying our sample text, replace any instances of XXX with the appropriate details.**<br><br>• If the data are **held or will be held in a public repository**, include URLs, accession numbers or DOIs. If this information will only be available after acceptance, indicate this by ticking the box below. For example: *All XXX files are available from the XXX database (accession number(s) XXX, XXX.*).<br>• If the data are all contained **within the manuscript and/or Supporting Information files**, enter the following: *All relevant data are within the manuscript and its Supporting Information files.*<br>• If neither of these applies but you are able to provide **details of access elsewhere**, with or without limitations, please do so. For example:<br><br>*Data cannot be shared publicly because of [XXX]. Data are available from the XXX Institutional Data Access / Ethics Committee (contact via XXX) for researchers who meet the criteria for access to confidential data.*<br><br>*The data underlying the results presented in the study are available from (include the name of the third party*	All relevant data are within the manuscript and its Supporting Information files.

and contact information or URL).<br>• This text is appropriate if the data are owned by a third party and authors do not have permission to share the data.<br><br>* typeset	
Additional data availability information:	

Manuscript Click here to access/download;Manuscript;Manuscript.docx

TITLE PAGE

A trial-based cost-effectiveness analysis of sugemalimab vs. placebo as consolidation therapy for unresectable stage III NSCLC in China

Wei Li*, Li Wan

Department of Pharmacy, Maternal and Child Health Hospital of Hubei Province, Tongji Medical College, Huazhong University of Science and Technology, 430070, Wuhan, China

*Corresponding author

E-mail: liwei1988hust@126.com (WL)

Abstract

Objective:

To assess the cost-effectiveness of sugemalimab vs. placebo in post-chemoradiotherapy patients with locally advanced, unresectable stage III NSCLC from the perspective of the Chinese healthcare system.

Methods:

A 3-state Markov model with a 3-week cycle length was performed to appraise the incremental cost-effectiveness ratio (ICER) of sugemalimab consolidation therapy based on the GEMSTONE-301 clinical trial (data cutoff March 1, 2022) over a 21-year time horizon. Costs and health utilities were obtained from local databases and published articles. Sensitivity and scenario analyses were adopted to evaluate the model uncertainty. Internal and external data sources were used to justify the plausibility of the extrapolated portion of the survival model chosen.

Results:

In comparison with the placebo, sugemalimab consolidation therapy was not cost-effective as it yielded an ICER value of $91,072 and $56,956 for the concurrent chemoradiotherapy (cCRT) and the sequential chemoradiotherapy (sCRT) population at the willingness-to-pay (WTP) threshold of $37,663/QALYs, respectively. When taking the sugemalimab patient assistance program (PAP) into consideration, sugemalimab consolidation therapy was cost-effective with an ICER dramatic decreases below the WTP. Sensitivity analysis demonstrated that the ICER was most sensitive to the discount rate and subsequent treatment. However, none of the sensitive parameters could affect the cost-effective conclusions without or with PAP. Scenario analysis revealed that the model was particularly affected by assumptions regarding discount in sugemalimab, choice of parametric survival models, mean duration and choice of subsequent immunotherapy.

Conclusions:

From the perspective of Chinese healthcare system, sugemalimab consolidation therapy was not a cost-effective strategy in cCRT and sCRT patients with unresectable stage III NSCLC. Given that the sugemalimab PAP was available, sugemalimab consolidation therapy became a cost-effective option.

1. Introduction

Lung cancer is the most common malignancy and the leading cause of tumor-related death in China (1). Non-small cell lung cancer (NSCLC) is the most prevalent subtype of lung cancer, and approximately 30% of newly diagnosed patients are at an advanced unresectable stage (stage III) (2). For a decade, platinum-based chemotherapy concurrent radiotherapy (cCRT) was the standard of care for patients with stage III NSCLC all over the world until the approval of durvalumab, which obtained satisfactory clinical benefit in PACIFIC trial (3,4). However, the PACIFIC trial restricted enrollment to patients after cCRT rather than sequential chemoradiotherapy (sCRT). As a matter of fact, due to tolerability and Eastern Cooperative Oncology Group (ECOG) performance status issues, many patients therefore chose sequential chemoradiotherapy (sCRT) as an option strategy (5, 6). As an alternative to cCRT, sCRT is also recommended in international treatment guidelines (7-9) and widely used in clinical practice throughout the world.

Sugemalimab (suge), a full length, fully human, anti-programmed death ligand 1 (PD-L1) monoclonal antibody, is being developed by CStone Pharmaceuticals. In the phase III GEMSTONE-301 trial (NCT03728556), sugemalimab vs. placebo significantly prolonged the median progression-free survival (PFS) and median overall survival (OS) (10). At data cutoff (March 1, 2022) for the final analysis (11, 12), the median PFS was 15.7 vs. 8.3 months (HR=0.71) and 8.1 vs. 4.1 months (HR=0.57) in cCRT and sCRT population, respectively. Furthermore, the median OS was not reached (NR) vs. 32.4 months (HR=0.75) and NR vs. 24.1 months (HR=0.60) in cCRT and sCRT population, respectively. Based on its significant clinical benefits and acceptable safety profile, sugemalimab was approved in China for consolidation treatment in patients with stage III NSCLC (13).

Despite its clinical benefits, the huge cost of sugemalimab consolidation therapy might bring potential economic ~~toxicity~~ to patients and their families, mirroring the efficiency issue in a health recourse-limited setting. Previous studies have assessed the cost-effectiveness of sugemalimab in first-line treatment of metastatic NSCLC (stage IV) (14, 15). However, the economic evaluation of sugemalimab therapy in different clinical setting (i.e. stage III NSCLC) remains to be performed. This study aimed to provide a comprehensive economic evaluation of sugemalimab consolidation therapy for unresectable stage III NSCLC in the context of the Chinese health care system.

2. Methods

2.1 Model structure and outcomes

A mathematical model that combined decision tree and Markov model was constructed to evaluate the clinical and economic outcomes of sugemalimab vs. placebo, as a consolidation therapy in patients with locally advanced, unresectable, stage III NSCLC (**Figure 1A**). A 3-state Markov model, including three mutually exclusive disease-related health states: PFS, progressed disease (PD), and death was performed by using TreeAge Pro 2011 (TreeAge Software, Williamstown, MA) (**Figure 1B**). It was assumed that all patients entered the model in the PFS state and then either remained in the same state or transited to the other states during a Markov cycle length of 3 weeks. The initial age of the simulated cohort population was set to 60 years old, which was in line with the average age of the GEMSTONE-301 trial. The time horizon for the model was about 21 years, which was close to the average life expectancy of China in 2021.

The primary model outcomes were the corresponding total costs of two therapeutic regimens, quality-adjusted life years (QALYs), and the incremental cost-effectiveness ratio (ICER). Both costs and utility

values were discounted at an annual rate of 5% for base-case analysis, according to guideline for health economic evaluations in China (16). All costs were converted into 2021 US dollars (US 1$ = CNY ¥6.45). Three times of the per capita gross domestic product (GDP) of China in 2021 (US$37,663/QALYs) was used as the willingness-to-pay (WTP) threshold to evaluate the cost-effectiveness of the two competing strategies (16).

2.2 Clinical data and transition probabilities

Clinical efficacy and safety data were obtained from GEMSTONE-301 trial (10) which recruited 381 Chinese patients between Aug 30, 2018 and Dec 30, 2020, were randomly assigned to sugemalimab (n=255) and to placebo (n=126). Patients received a continuous and fixed dose of sugemalimab (1200mg, q3w) or placebo as consolidation therapy for up to 24 months, unless confirmed disease progression, unacceptable toxicity or withdrawal of consent.

The time-dependency transition probabilities of patients remaining in PFS and survival state were derived from the PFS and OS Kaplan-Meier (K-M) data of the GEMSTONE-301 trial, data cutoff at March 1, 2022, respectively (11, 12). First, GetData Graph Digitizer software (version 2.26) was employed to extracted the graphical data from published K-M curves. Second, R software (version 4.0.3) was used to reconstruct the individual patient data (IPD) derived by Hoyle et al (17). Third, to extrapolate survival curve beyond the observation period, we considered the following two-parametric distributions to fit the IPD: Exponential, Weibull, Gompertz, Log-logistic, and Log-normal. The choice of the model to use was based on Akaike's information criterion (AIC), Bayesian information criterion (BIC), visual inspection and clinical plausibility of the extrapolations. Finally, the best fitting distribution parameters in both two arms were obtained by using the following survival function (Exponential: $S(t) = e^{(-\lambda t)}$,

Weibull: $S(t) = e^{(-\lambda t^{\gamma})}$, Log-logistics: $S(t) = 1/[(1 + (\lambda t)^{\gamma}]$, Log-normal: $S(t) = \frac{1}{2} - \frac{1}{2} erf\left[\frac{\sqrt{2}(\ln(t) - \lambda)}{2\gamma}\right]$, Gompertz: $S(t) = e^{\left[\frac{\lambda}{\gamma}(1 - e^{(\gamma t)})\right]}$ and the best fitting for PFS and OS curves were shown in Table 1 and **Tables S1**. We also provided the exploration and fitting of PFS and OS curves for visual inspection (see **Figures 2, 3**). To calculate the probability of transition from the PFS state to death state, we assumed that it was equal to the age-specific background mortality derived from the National Bureau of Statistics of China (2019) (18). The method for calculating transition probabilities was detailed in the published article (19).

Table 1. Best fit and the values of the parameters.

	best fitting	Scale (λ)	Shape (γ)
**cCRT population**			
Suge, OS	Log-normal	4.05400	1.20300
Suge, PFS	Gompertz	0.04322	-0.03176
Placebo, OS	Log-normal	3.78600	1.09100
Placebo, PFS	Gompertz	0.05935	-0.03300
**sCRT population**			
Suge, OS	Gompertz	0.00735	0.01470
Suge, PFS	Gompertz	0.07675	-0.04160
Placebo, OS	Weibull	0.00171	1.64869
Placebo, PFS	Gompertz	0.14236	-0.04570

PFS, progression-free survival; OS, overall survival; cCRT, concurrent chemoradiotherapy; sCRT, sequential chemoradiotherapy; Suge, sugemalimab.

2.3 Cost and utility

Only direct medical costs, calculated from the perspective of the Chinese healthcare system, including costs of drug (maintenance and subsequent treatment), and management of serious adverse events (SAEs, grade 3 to 4), routine follow-up, terminal care in end of life. In calculating dosage amounts, we assumed a 60-year-old patient with an average weight of 65Kg, height of 1.64 m, and 70 mL/min creatinine clearance (20), resulting in a body surface area (BSA) of 1.72 m^2 (21). Currently, sugemalimab patient

assistance program (PAP) was conducted for eligible patients to improve the drug affordability in China (22). The PAP supports patients to pay for 2 cycles of sugemalimab, followed by 2 cycles of free sugemalimab (2+2); and then pay for 2 cycles, followed by 25 cycles of donations (2+25); and then pay for 1 cycle, followed by 3 cycles of donations (1+3).

At progression, patients would receive platinum-based chemotherapy, with or without immunotherapy. According to the interim analysis (cutoff date: March 8, 2021) of GEMSTONE-301 trial, sintilimab and pembrolizumab were the preferred option for subsequent immunotherapy (10). Because of the large price difference between the two drugs, we used sintilimab for the baseline analysis and pembrolizumab for the scenario analysis. As no update detailed subsequent anticancer treatment data were shown for GEMSTONE-301 trial at cutoff date March 1, 2022, we used the update 4-year survival data of the PACIFIC Trial (23). In PACIFIC trial, the percentage of immunotherapy at progression for durvalumab arm and placebo arm was 24.5% and 48.6%, respectively. For simplification, we assumed that patients with non-squamous (NSQ) NSCLC after progression to receive sintilimab (200mg, q3w) or placebo, plus carboplatin (AUC 5 mg/mL/min, q3w) and pemetrexed (500 mg/m^2, q3w), for up to 4 cycles, followed by sintilimab or placebo for up to a total of 35 cycles plus pemetrexed maintenance therapy (24). Patients with squamous (SQ) NSCLC after progression to receive sintilimab (200mg, q3w) or placebo, plus carboplatin (AUC 5 mg/mL/min, q3w) and gemcitabine (1.0 g/m^2, day 1 and day 8, q3w), for up to 4 cycles, followed by sintilimab or placebo for up to a total of 35 cycles maintenance therapy (25). In addition, a 2-year maximum treatment duration of sugemalimab, sintilimab and pembrolizumab was taken into consideration based on previous studies (26). Due to the low incidence of SAEs (less than 2% in both arms), we did not consider the costs and disutility values related to SAEs. All costs were derived

from local charge, median price of the winning bid product derived from the Chinese Drug Bidding Database by the YaoZH (27) or previously published literature.

Health utility values for each health state in our model were derived from previously published studies, as no health-related quality of life (HRQoL) was published in clinical trial. We set the health utility values estimates for PFS and PD was 0.901 and 0.863, respectively (28). Furthermore, a half-cycle correction was implemented to the outcomes, according to the ~~TreeAge Pro 2011 manual and~~ China Guidelines for Pharmacoeconomic Evaluation (16).

2.4 Sensitivity analysis

One-way deterministic (DSA) and probabilistic sensitivity analysis (PSA) were performed to assess the robustness of model outcomes. In one-way sensitivity analysis, relevant parameters that had substantial impact on ICER, were tested orderly at the upper and lower limits of plausible ranges, which were listed and illustrated in **Table 2**. The results of one-way sensitivity analysis were graphed in the tornado diagram. PSA was performed to determine the effects of uncertainty in all model parameters simultaneously via 1000 Monte Carlo simulations, which were illustrated in cost-effectiveness acceptability curve. The key parameters input our model were shown in **Table 1** and **Table 2**.

Table 2. Model inputs: base case values, ranges, and distributions for sensitivity analysis.

Parameters	Base case	Range	Distribution	Source
**Costs (US $)**				
Sugemalimab (600mg)	1,918.6	Fixed	Fixed in PSA	YaoZH
Carboplatin (100mg)	8.0	6.4-9.6	Gamma	Local charge

Pemetrexed (500mg)	424.2	339.4-509.0	Gamma	YaoZH
Pembrolizumab (100 mg)	2778	2222.4-3333.6	Gamma	YaoZH
Sintilimab (100mg)	440.8	352.6-529.0	Gamma	YaoZH
Gemcitabine (200mg)	6.6	5.3-7.9	Gamma	Local charge
Routine follow-up cost per cycle	55.6	27.8-83.4	Gamma	(21)
Terminal care per cycle	2,627.8	1,313.9-3,941.7	Gamma	(21)
**Utility values**				
Utility of PFS	0.901	0.883-0.918	Beta	(28)
Utility of PD	0.863	0.845-0.880	Beta	(28)
Discount rate(%)	5	0-8	Fixed in PSA	(16)
**others**				
Proportion of subsequent immunotherapy for suge arm	0.245	0-0.245	Beta	(23)
Proportion of subsequent immunotherapy for placebo arm	0.486	0.486-1	Beta	(23)
Weight (kg)	65	52-78	Normal	(21)
Body surface area (m^2)	1.72	1.38-2.06	Normal	(21)

PFS, progression-free survival; PD, progressed disease; PSA, probabilistic sensitivity analysis; suge, sugemalimab.

2.5 Scenario analysis

Scenario analysis were performed using variations in key model setting and assumptions, including time horizon, tumor histological type distribution, discount in sugemalimab, choice of subsequent immunotherapy, mean duration of maintenance treatment and subsequent immunotherapy. For the immature data, especially in the OS, the Weibull models were used in scenario analysis to explore the impact brought by difference survival models. The parameter values of Weibull distribution for PFS and OS curves were exhibited in **Table S2**.

3. Results

3.1 Base-case analysis

Discounted results for sugemalimab following cCRT or sCRT vs. placebo were presented in Table 3. Compared with the placebo arm, suge arm resulted in ICER values of $91,072 and $56,956 for the cCRT and sCRT population, respectively (**Table 3**). The suge arm was clearly not a cost-effective strategy for the cCRT and sCRT population under the Chinese cost-effectiveness WTP threshold of $37,663/QALYs, to receive maintenance therapy with sugemalimab, in comparison of placebo. However, when the PAP was considered, the suge arm became a cost-effective strategy for both cCRT and sCRT populations (see **Table 3**).

Table 3. Base-case analysis results without or with PAP.

Population	Treatment	Cost ($)	Incremental cost	QALYs	Incremental QALYs	ICER
cCRT						
without PAP	suge	83,146	73,768	3.90	0.81	91,072
	placebo	9,378	NA	3.09	NA	NA
with PAP	suge	20,969	11,591	3.90	0.81	14,310
	placebo	9,378	NA	3.09	NA	NA
sCRT						
without PAP	suge	66,317	52,969	2.95	0.93	56,956
	placebo	13,348	NA	2.02	NA	NA
with PAP	suge	20,778	7,430	2.95	0.93	7,989
	placebo	13,348	NA	2.02	NA	NA

cCRT, concurrent chemoradiotherapy; sCRT, sequential chemoradiotherapy; QALYs, quality-adjusted life years; ICER, incremental cost-effectiveness ratio; NA, not applicable; suge, sugemalimab.

3.2 DSA

Tornado diagrams of the top 13 most influential key variables in cCRT and sCRT population were illustrated in Figure 4. According to the one-way DSA results, the discount rate, proportion of patients received subsequent immunotherapy in placebo or suge arm after disease progression were the main

driving variables. However, none of the fluctuations of sensitive parameters could affect the cost-effective conclusions without (**Figure 4A, 4B**) or with PAP (**Figure 4C, 4D**) for cCRT and sCRT population. On the whole, our base-case analysis results were relatively robust.

3.3 PSA

The results of PSA were presented in **Figure 5**. The results showed that, under the chosen WTP ($37,663/QALYs), the probability of the suge arm being cost-effective compared with the placebo arm was 0% for both cCRT and sCRT population. However, when the WTP threshold increased to $56,967/QALYs, the probability of suge arm being economical was 0 and 50% for the cCRT and sCRT population, respectively. Given that the PAP was available, the probability of the suge arm being a cost-effective strategy was 100%.

3.4 Scenario analysis results

The results of each scenario analysis for cCRT and sCRT populations were displayed in **Table 4** and **Table 5**, respectively. Scenario analysis for cCRT population revealed that the model was most sensitive to assumptions regarding time horizon ($91,072-133,495), tumor histological type distribution ($89,964-91,547), discount in sugemalimab ($53,749-72,411), choice of subsequent immunotherapy ($75,740), mean duration of maintenance treatment ($31,570-55,081), mean duration of subsequent immunotherapy ($92,889-93,851) and choice of Weibull survival models ($107,664) (**Table 4**). However, no scenario could drag the ICER below the $37,663 WTP threshold except for the mean duration of maintenance treatment. Furthermore, the ICER of suge arm remained surpass $37,663 except for scenarios that the discount in sugemalimab, choice of subsequent immunotherapy and the mean duration of maintenance treatment for the sCRT population (**Table 5**).

Table 4. Results of scenario analysis for cCRT population.

Scenario	Cost suge	Cost placebo	Incremental cost	QALYs suge	QALYs placebo	Incremental QALYs	ICER
**Time horizon**							
10-y	82,832	9,410	73,422	3.38	2.83	0.55	133,495
15-y	83,099	9,391	73,708	3.72	3.01	0.71	103,814
21-y (base case)	83,146	9,378	73,768	3.90	3.09	0.81	91,072
**Tumor histological type**							
100% SQ	81,522	7,369	74,153	3.90	3.09	0.81	91,547
100% NSQ	86,935	14,064	72,871	3.90	3.09	0.81	89,964
**Discount in suge**							
80%	68,031	9,378	58,653	3.90	3.09	0.81	72,411
60%	52,915	9,378	43,537	3.90	3.09	0.81	53,749
**Choice of subsequent immunotherapy**							
Pembrolizumab	91,683	30,334	61,349	3.90	3.09	0.81	75,740
**Mean duration of maintenance treatment**							
6 mo	34,950	9,378	25,572	3.90	3.09	0.81	31,570
12 mo	53,994	9,378	44,616	3.90	3.09	0.81	55,081
**Mean duration of subsequent immunotherapy**							
6 mo	80,382	4,363	76,019	3.90	3.09	0.81	93,851
12 mo	81,235	5,995	75,240	3.90	3.09	0.81	92,889
**Survival curves options**							
Weibull	83,666	8,301	75,365	2.68	1.98	0.70	107,664

cCRT, concurrent chemoradiotherapy; sCRT, sequential chemoradiotherapy; QALYs, quality-adjusted life years; ICER, incremental cost-effectiveness ratio; suge, sugemalimab.

Table 5. Results of scenario analysis for sCRT population.

Scenario	Cost suge	Cost placebo	Incremental cost	QALYs suge	QALYs placebo	Incremental QALYs	ICER
**Time horizon**							
10-y	66,332	13,349	52,983	2.93	2.02	0.91	58,223
15-y	66,317	13,348	52,969	2.94	2.02	0.92	57,575

21-y (base case)	66,317	13,348	52,969	2.95	2.02	0.93	56,956
**Tumor histological type**							
100% SQ	63,484	9,622	53,862	2.95	2.02	0.93	57,916
100% NSQ	72,925	22,044	50,881	2.95	2.02	0.93	54,711
**Discount in suge**							
80%	54,825	13,348	41,477	2.95	2.02	0.93	44,599
60%	43,333	13,348	29,985	2.95	2.02	0.93	32,242
**Choice of subsequent immunotherapy**							
Pembrolizumab	81,203	52,266	28,937	2.95	2.02	0.93	31,115
**Mean duration of maintenance treatment**							
6 mo	33,303	13,348	19,955	2.95	2.02	0.93	21,457
12 mo	47,466	13,348	34,118	2.95	2.02	0.93	36,686
**Mean duration of subsequent immunotherapy**							
6 mo	61,458	4,244	57,214	2.95	2.02	0.93	61,520
12 mo	62,909	7,391	55,518	2.95	2.02	0.93	59,697
**Survival curves options**							
Weibull	70,696	13,165	57,531	3.52	2.01	1.51	38,100

cCRT, concurrent chemoradiotherapy; sCRT, sequential chemoradiotherapy; QALYs, quality-adjusted life years; ICER, incremental cost-effectiveness ratio; suge, sugemalimab.

3.5 Model validation

Validation results for modeled PFS and OS data are presented in **Table 6**. The mPFS, mOS, and 1,2,3,5-year survival rate estimated by best-fitting distribution models matched the results that observed in GEMSTONE-301(cutoff date: March 1, 2022) (11, 12), PACIFIC (23) and START (29) clinical trials satisfactorily.

Table 6. Validation of modeled PFS and OS data using updated GEMSTONE-301 and external data.

Curve	Median (month)	1-year (%)	2-year (%)	3-year (%)	5-year (%)
**cCRT population**					
**PFS**					
Suge arm					
Modeled	15.4	56.7	40.7	33.6	28.1
GEMSTONE-301	15.7	54.8	42.5	34.1	NA
PACIFIC	17.2	55.3	44.8	39.8	28.2
Placebo arm					
Modeled	10.2	46.2	29.7	23.1	18.5
GEMSTONE-301	8.3	42.1	34.0	NA	NA
PACIFIC	5.6	34.4	24.8	20.5	NA
**OS**					
Suge arm					
Modeled	40.6	84.5	67.0	54.0	37.3
GEMSTONE-301	NR (28.2, NR)	85.4	66.3	54.1	NA
PACIFIC	47.5	83.1	66.3	56.7	42.1
Placebo arm					
Modeled	30.8	80.9	59.4	44.7	27.4
GEMSTONE-301	32.4 (20.6, NR)	84.8	57.6	19.8	NA
PACIFIC	29.1	74.6	55.3	43.6	32.7
**sCRT population**					
**PFS**					
Suge arm					
Modeled	7.7	39.2	24.7	19.7	16.7
GEMSTONE-301	8.1	38.8	30.5	18.1	NA
Placebo arm					
Modeled	4.0	18.6	8.6	6.0	4.7
GEMSTONE-301	4.1	12.7	7.6	NA	NA
**OS**					
Suge arm					
Modeled	41.3	86.8	72.3	57.2	28.8
GEMSTONE-301	NR (31.9, NR)	88.4	70.7	59.0	NA
Placebo arm					
Modeled	26.6	83.3	56.3	32.6	7.4
GEMSTONE-301	24.1	80.2	53.7	43.7	NA
START	24.6	74.4	50.9	37.7	about 9.8

cCRT, concurrent chemoradiotherapy; sCRT, sequential chemoradiotherapy; mPFS, median progression-free survival; mOS, median overall survival; NR: not reached; Suge, sugemalimab.

4. Discussion

Till now, a number of trial-based economic assessments on durvalumab in post-chemoradiotherapy patients with unresectable stage III NSCLC from the Chinese healthcare system perspective have been reported (28, 30, 31). The clinical data of these studies were all acquired from the 4-year survival updated PACIFIC clinical trial (23). Sugemalimab, as the second domestic PD-L1 inhibitor approved by China for treating unresectable stage III NSCLC, represented a milestone for Chinese patients suffering from lung cancer. However, the high costs of this novel therapy might bring financial toxicity for the majority of middle- and low-income rural patients. It is an urgent agenda to evaluate the cost-effectiveness of sugemalimab consolidation therapy for lung cancer in the Chinse context. To the best of our knowledge, this is the first economic study on sugemalimab consolidation therapy for unresectable stage III NSCLC in China.

In this present study, we built a dynamic state transition model based on the latest data from Asian ethnicity population in GEMSTONE-301 study to estimate the cost-effectiveness of sugemalimab vs. placebo as consolidation therapy for unresectable stage III NSCLC in China. And we found that sugemalimab consolidation therapy was not cost-effective compared with placebo for sCRT and cCRT population at current WTP threshold ($37,663/QALYs). The economic probability of suge arm for cCRT and sCRT population increased with the raise of WTP, but the sCRT was more likely to be economic at a relatively lower threshold. However, when considering the price reduction (i.e. PAP), the suge arm exhibited favorable cost-effectiveness for both cCRT and sCRT population. The results of one-way deterministic and probabilistic sensitivity analysis both proved that the outcomes were generally robust.

Due to the immature of OS data in GEMSTONE-301, poorly parametric model selection might overestimate or underestimate long-term survival outcomes beyond the study follow-up period. To

minimize the potential bias introduced by model selection, we introduced both internal and external validations. The selection of best-fitting parametric distributions for PFS and OS curves in each arm was the result of a comprehensive analysis, taking into account not only the AIC, BIC and median survival time but also the long-term survival rate, as well as clinical rationality. The Gompertz distribution model for PFS curve in placebo arm estimated a mPFS of 10.2 month for cCRT population, which seemed to be a significant difference comparing with that of the 8.3 month in GEMSTONE-301. Judging only by the mPFS, the Gompertz distribution model did not seem to be the best fit choice. However, the 5-year survival rate in placebo arm of cCRT population estimated by Gompertz model (18.5 vs. <19.5%) was closest to that of PACIFIC clinical trial (i.e. external data) among all distribution models. The Log-normal model estimated a 5-year survival rate of 37.3 vs. 27.4% for suge arm and placebo arm in cCRT, which was approximate to that of PACIFIC trial (42.1 vs. 32.7%). By comparing the baseline characteristics of GEMSTONE-301 and PACIFIC (3), it was not difficult to conclude that the GEMSTONE-301 study population was more refractory, with higher proportion of patients with stage IIIB/IIIC, squamous cell carcinoma, and ECOG PS=1, resulting a relatively lower survival rate. For sCRT population, the 5-year overall survival rate in placebo arm was 7.4%, which was close to that of about 9.8% in START trial (29). As for the suge arm in sCRT, the estimated 5-year survival rate was 28.8%, lower than that of 37.3% in cCRT, which was in line with the conclusion of a previously reported Meta-analysis (32). As NICE DSU TSD 14 recommended (33) that the use of the same type of parametric model for individual intervention groups to evite drastically different shapes of survival curves, unless there is a reasonable explanation. Although we selected different types of parametric models for individual treatment arms, deviating from this guidance, this selection exhibited superior external and

internal validity and plausibility. In addition, we also chose one parametric model (i.e. Weibull) to fit the entire dataset in scenario analysis to explore the model stability.

There were several limitations in this study that should to be noted. Firstly, the state-transition model used in this present study was that PFS, OS, and OS data were immature, which might affect the simulation of the long-term survival curves and increase model uncertainty. Secondly, the lack of HRQoL data from China, which has different patient preferences and healthcare settings from many developed countries, might bring bias in economic evaluations. Thirdly, while the base-case analysis represented a likely set of therapeutic regimens in China, in clinical practice, different patients might have different preferences for subsequent treatment options. Despite the limitations of this present study, the uncertainty analysis demonstrated the robustness of our model outcomes. Therefore, it might provide valuable reference information for clinical treatment decisions and policy-makers.

5. Conclusions

From the perspective of Chinese healthcare system, sugemalimab consolidation therapy vs. placebo after cCRT or sCRT was not cost-effective for patients with unresectable stage III NSCLC. However, the use of the Patient Assistance Program (PAP) should be considered as a favorable cost-effective treatment option in China.

Supporting information

Table S1. AIC and BIC of 5 parametric distributions for cCRT and sCRT curves.

Table S2. Parameter values of Weibull distribution.

Financial Disclosure

This work was supported by the Maternal and Child Health Hospital of Hubei Province Research Project

(No. 2021SFYM030). The funders had no role in study design, data collection and analysis, decision to publish, or preparation of the manuscript.

Author Contributions

Investigation: Wei Li, Li Wan. Methodology: Wei Li**.** Software: Li Wan**.** Writing – original draft: Wei Li**.** Writing – review & editing: Wei Li, Li Wan.

Competing Interests

The authors have declared that no competing interests exist.

Ethics Statement: N/A.

Data availability statement: All data generated or analyzed during this study are included in this manuscript and its supplementary materials.

References

1. Sung H, Ferlay J, Siegel RL, et al. Global Cancer Statistics 2020: GLOBOCAN Estimates of Incidence and Mortality Worldwide for 36 Cancers in 185 Countries. CA Cancer J Clin. 2021;71(3):209-49. doi: 10.3322/caac.21660

2. Morgensztern D, Ng SH, Gao F, et al. Trends in stage distribution for patients with non-small cell lung cancer: a National Cancer Database survey. J Thorac Oncol. 2010;5(1):29-33. doi: 10.1097/JTO.0b013e3181c5920c

3. Antonia SJ, Villegas A, Daniel D, et al. Overall Survival with Durvalumab after Chemoradiotherapy in Stage III NSCLC. N Engl J Med. 2018;379(24):2342-50. doi: 10.1056/NEJMoa1809697

4. Curran WJ, Jr., Paulus R, Langer CJ, et al. Sequential vs. concurrent chemoradiation for stage III non-small cell lung cancer: randomized phase III trial RTOG 9410. J Natl Cancer Inst. 2011;103(19):1452-60. doi: 10.1093/jnci/djr325

5. Conibear J, AstraZeneca UKL. Rationale for concurrent chemoradiotherapy for patients with stage III non-small-cell lung cancer. Br J Cancer. 2020;123(Suppl 1):10-7. doi: 10.1038/s41416-020-01070-6

6. Zhu D, Ding R, Ma Y, et al. Comorbidity in lung cancer patients and its association with hospital readmission and fatality in China. BMC Cancer. 2021;21(1):557. doi: 10.1186/s12885-021-08272-y

7. Ettinger DS, Wood DE, Aisner DL, et al. NCCN Guidelines Insights: Non-Small Cell Lung Cancer, Version 2.2021. J Natl Compr Canc Netw. 2021;19(3):254-66. doi: 10.6004/jnccn.2021.0013

8. Postmus PE, Kerr KM, Oudkerk M, et al. Early and locally advanced non-small-cell lung cancer (NSCLC): ESMO Clinical Practice Guidelines for diagnosis, treatment and follow-up. Ann Oncol. 2017;28(suppl_4):iv1-iv21. doi: 10.1093/annonc/mdx222

9. Park K, Vansteenkiste J, Lee KH, et al. Pan-Asian adapted ESMO Clinical Practice Guidelines for the management of patients with locally-advanced unresectable non-small-cell lung cancer: a KSMO-ESMO initiative endorsed by CSCO, ISMPO, JSMO, MOS, SSO and TOS. Ann Oncol. 2020;31(2):191-201. doi: 10.1016/j.annonc.2019.10.026

10. Zhou Q, Chen M, Jiang O, et al. Sugemalimab versus placebo after concurrent or sequential chemoradiotherapy in patients with locally advanced, unresectable, stage III non-small-cell lung cancer in China (GEMSTONE-301): interim results of a randomised, double-blind, multicentre, phase 3 trial. Lancet Oncol. 2022;23(2):209-19. doi: 10.1016/S1470-2045(21)00630-6

11. Final progression-free survival analysis of GEMSTONE-301study. Available online at: https://mp.weixin.qq.com/s/7yd_1sZbQea3LfO3WCgPPA (accessed Oct 10, 2022).

12. Wu Y, Zhou Q, Chen M, et al. Sugemalimab vs Placebo after cCRT or sCRT in pts with Unresectable Stage III NSCLC: Final PFS Analysis of a Phase 3 Study. 2022 WCLC, OA02.05.

13. Approval of sugemalimab in patients with unresectable stage III non-small cell lung cancer in China. (2022). Available online at: https://www.cstonepharma.com/en/html/news/2788.html (accessed Oct 10, 2022).

14. Li W, Wan L. Cost-effectiveness analysis of sugemalimab vs. placebo, in combination with chemotherapy, for treatment of first-line metastatic NSCLC in China. Front Public Health. 2022;10: 1015702. doi: 10.3389/fpubh.2022.1015702

15. Zheng Z, Zhu H, Fang L, et al. Cost-effectiveness analysis of sugemalimab vs. chemotherapy as first-line treatment of metastatic nonsquamous non-small cell lung cancer. Front Pharmacol. 2022;13:996914. doi: 10.3389/fphar.2022.996914

16. Chinese Pharmaceutical Association. China Guidelines for Pharmacoeconomic Evaluations. (2019). Available online at: https://www.cpa.org.cn//?do=infoandcid=75017 (accessed May 11, 2022).

17. Hoyle MW, Henley W. Improved curve fits to summary survival data: application to economic evaluation of health technologies. BMC Med Res Methodol. 2011;11:139. doi: 10.1186/1471-2288-11-139

18. National Bureau of Statistics of China. China Population and Employment Statistic Yearbook 2019. Beijing: China Statistic Press (2019).

19. Rui M, Shi F, Shang Y, et al. Economic Evaluation of Cisplatin Plus Gemcitabine Versus Paclitaxel Plus Gemcitabine for the Treatment of First-Line Advanced Metastatic Triple-Negative Breast Cancer in China: Using Markov Model and Partitioned Survival Model. Adv Ther. 2020;37(9):3761-74. doi: 10.1007/s12325-020-01418-7

20. Wang S, Peng L, Li J, et al. A trial-based cost-effectiveness analysis of erlotinib alone versus platinum-based doublet chemotherapy as first-line therapy for Eastern Asian nonsquamous non-small-cell lung cancer. PLoS One. 2013;8(3):e55917. doi: 10.1371/journal.pone.0055917

21. Qiao L, Zhou Z, Zeng X, et al. Cost-Effectiveness of Domestic PD-1 Inhibitor Camrelizumab Combined With Chemotherapy in the First-Line Treatment of Advanced Nonsquamous Non-Small-Cell Lung Cancer in China. Front Pharmacol. 2021;12:728440. doi: 10.3389/fphar.2021.728440

22. Sugemalimab Patient Assistance Program. (2022). Available online at: https://mp.weixin.qq.com/s/xIzB3Gmb5M5OnG8qixyTtQ (accessed Oct 10, 2022).

23. Faivre-Finn C, Vicente D, Kurata T, et al. Four-Year Survival With Durvalumab After Chemoradiotherapy in Stage III NSCLC-an Update From the PACIFIC Trial. J Thorac Oncol. 2021;16(5):860-7. doi: 10.1016/j.jtho.2020.12.015

24. Yang Y, Wang Z, Fang J, et al. Efficacy and Safety of Sintilimab Plus Pemetrexed and Platinum as First-Line Treatment for Locally Advanced or Metastatic Nonsquamous NSCLC: a Randomized,

Double-Blind, Phase 3 Study (Oncology pRogram by InnovENT anti-PD-1-11). J Thorac Oncol. 2020;15(10):1636-46. doi: 10.1016/j.jtho.2020.07.014

25. Zhou C, Wu L, Fan Y, et al. Sintilimab Plus Platinum and Gemcitabine as First-Line Treatment for Advanced or Metastatic Squamous NSCLC: Results From a Randomized, Double-Blind, Phase 3 Trial (ORIENT-12). J Thorac Oncol. 2021;16(9):1501-11. doi: 10.1016/j.jtho.2021.04.011

26. Wu B, Zhang Q, Sun J. Cost-effectiveness of nivolumab plus ipilimumab as first-line therapy in advanced renal-cell carcinoma. J Immunother Cancer. 2018;6(1):124. doi: 10.1186/s40425-018-0440-9

27. The big data service platform for China's health industry. Information Query Drug Bid Winning. (2022). Available online at: https://www.yaozh.com/ (accessed Oct 10, 2022).

28. Jiang X, Chen J, Zheng M, et al. Cost-effectiveness analysis of durvalumab as a maintenance treatment for patients with locally advanced, unresectable, stage nsclc in china. PLoS One. 2022;17(6):e0270118. doi: 10.1371/journal.pone.0270118

29. Butts C, Socinski MA, Mitchell PL, et al. Tecemotide (L-BLP25) versus placebo after chemoradiotherapy for stage III non-small-cell lung cancer (START): a randomised, double-blind, phase 3 trial. Lancet Oncol. 2014;15(1):59-68. doi: 10.1016/S1470-2045(13)70510-2

30. Shi J, Ran Q, Gao S, et al. Cost- utility analysis of durvalumab for consolidation therapy after chemoradiotherapy for stage III non-small cell lung cancer. China Pharmacy. 2022;33(15):1860-4. doi:10.6039/j.issn.1001-0408.2022.15.13

31. Chen X, Yang Z, Xiang G, et al. Durvalumab consolidation therapy in patients with stage III non-small cell lung cancer after concurrent chemoradiation: a China-based cost-effectiveness analysis. Expert Rev Pharmacoecon Outcomes Res. 2022;22(4):647-54. doi: 10.1080/14737167.2022.1993062

32. Auperin A, Le Pechoux C, Rolland E, et al. Meta-analysis of concomitant versus sequential radiochemotherapy in locally advanced non-small-cell lung cancer. J Clin Oncol. 2010;28(13):2181-90. doi: 10.1200/JCO.2009.26.2543

33. Latimer NR. Survival Analysis For Economic Evaluations Alongside Clinical Trials - Extrapolation with Patient-Level Data. NICE Decision Support Unit Technical Support Documents. London2013. doi: 10.1177/0272989X12472398

Figure Legends

Figure 1: The schematics of the decision tree (A) and the Markov state transition model (B).

Figure 2: The exploration and fitting of PFS and OS curves for cCRT population. Model simulation visual PFS curve (A), OS curve (B) of sugemalimab arm and PFS curve (C), OS curve (D) of placebo arm.

Figure 3: The exploration and fitting of PFS and OS curves for sCRT population. Model simulation visual PFS curve (A), OS curve (B) of sugemalimab arm and PFS curve (C), OS curve (D) of placebo arm.

Figure 4: Tornado diagram of one-way deterministic sensitivity analysis (DSA) of sugemalimab vs. placebo. DSA for cCRT (A) and sCRT (B) population without PAP; DSA for cCRT (C) and sCRT (D) population with PAP.

Figure 5: Cost-effectiveness acceptability curves of sugemalimab vs. placebo with and without PAP.

Figure 1

Click here to access/download;Figure;Figure 1.tif

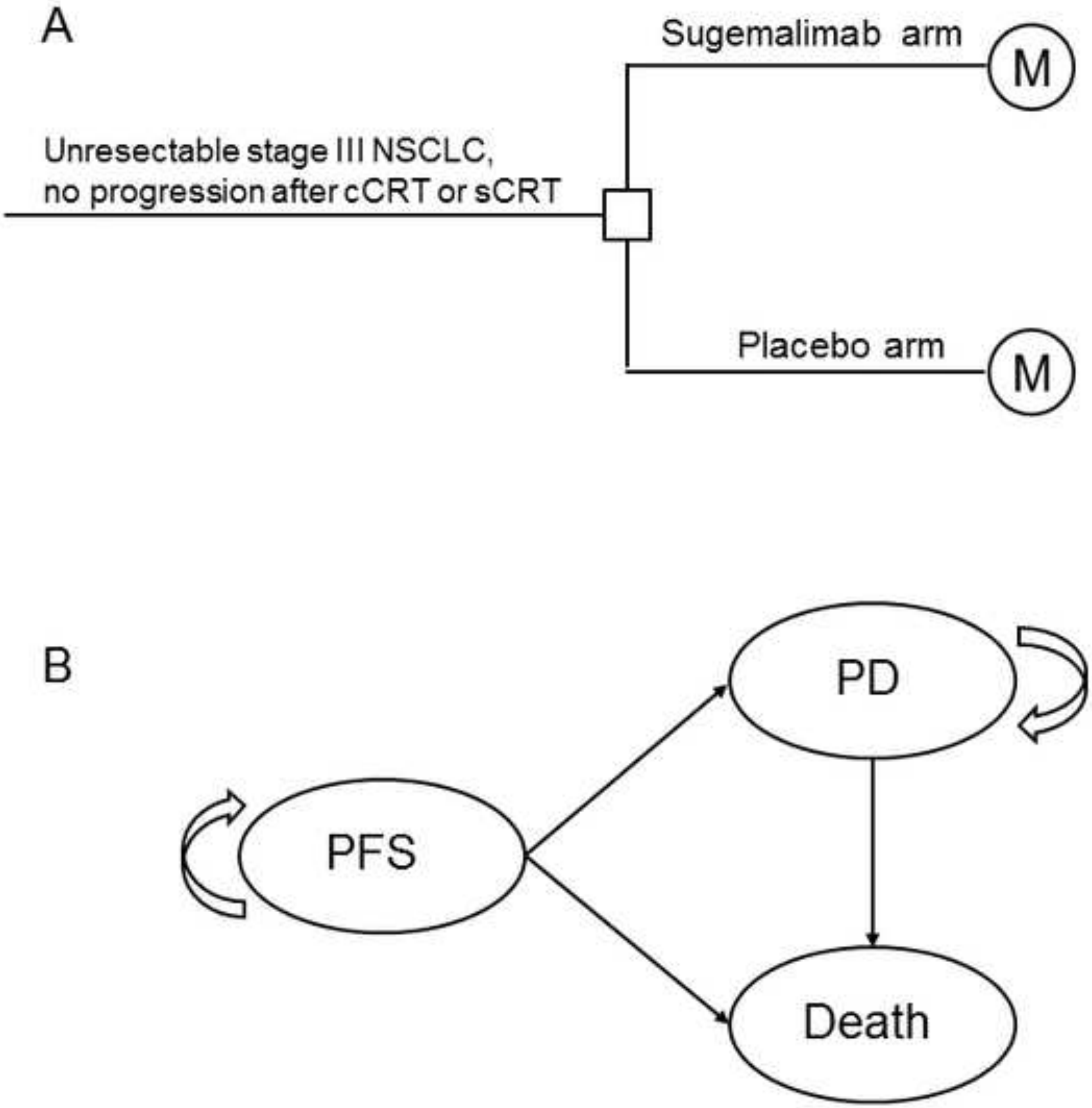

Figure 2

Click here to access/download;Figure;Figure 2.tif

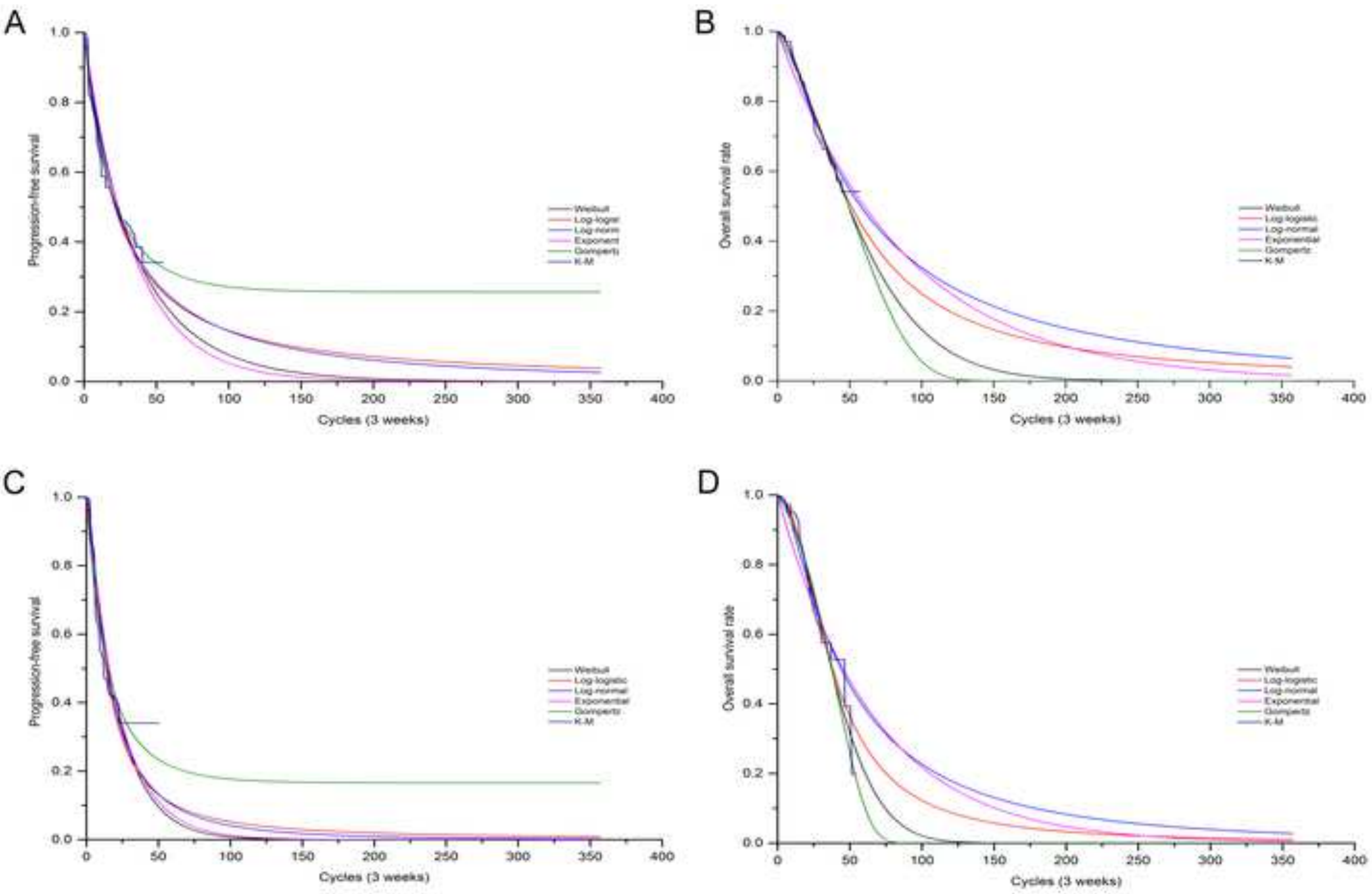

Figure 3

Click here to access/download;Figure;Figure 3.tif

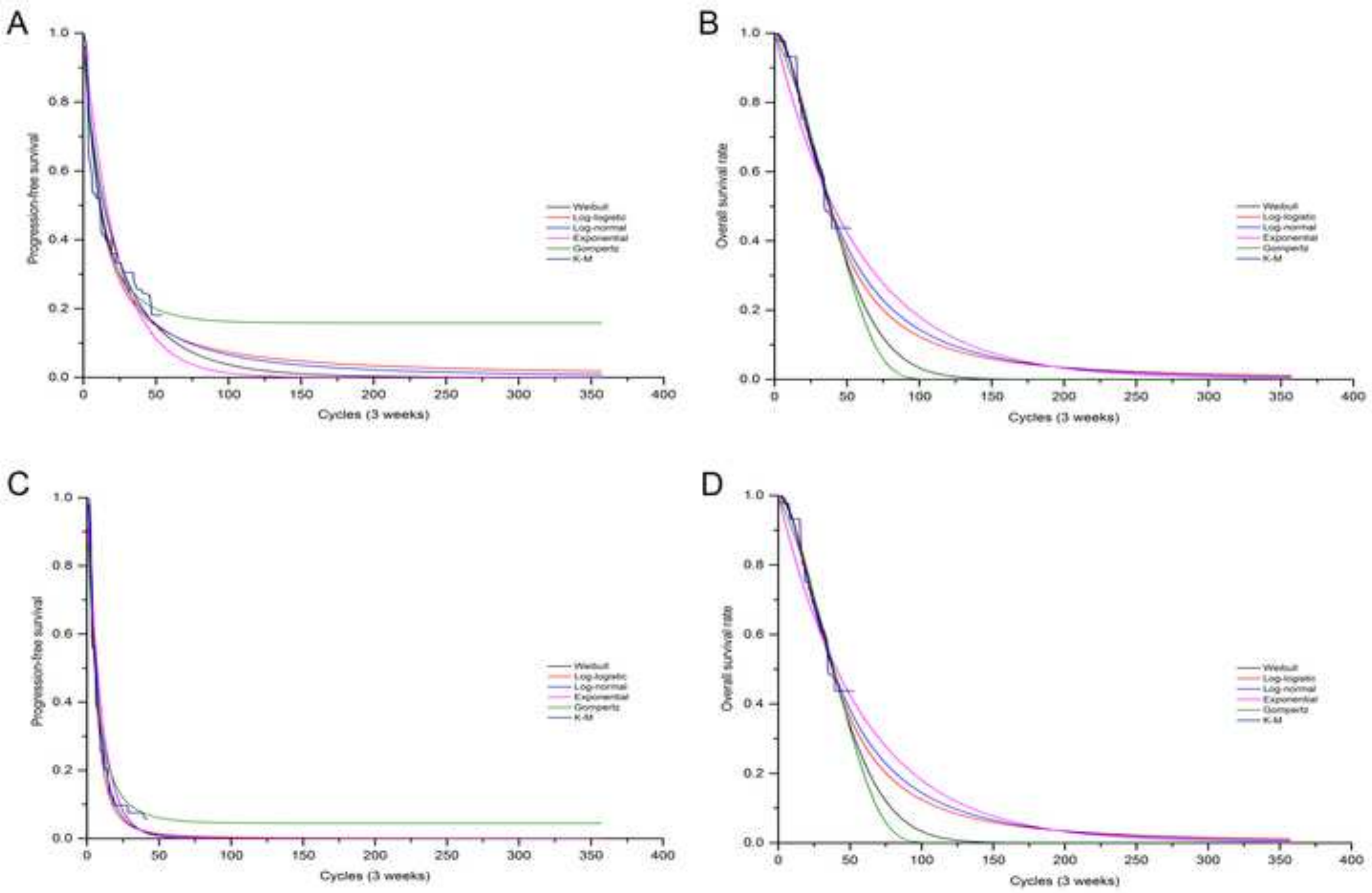

Figure 4

Click here to access/download;Figure;Figure 4.tif

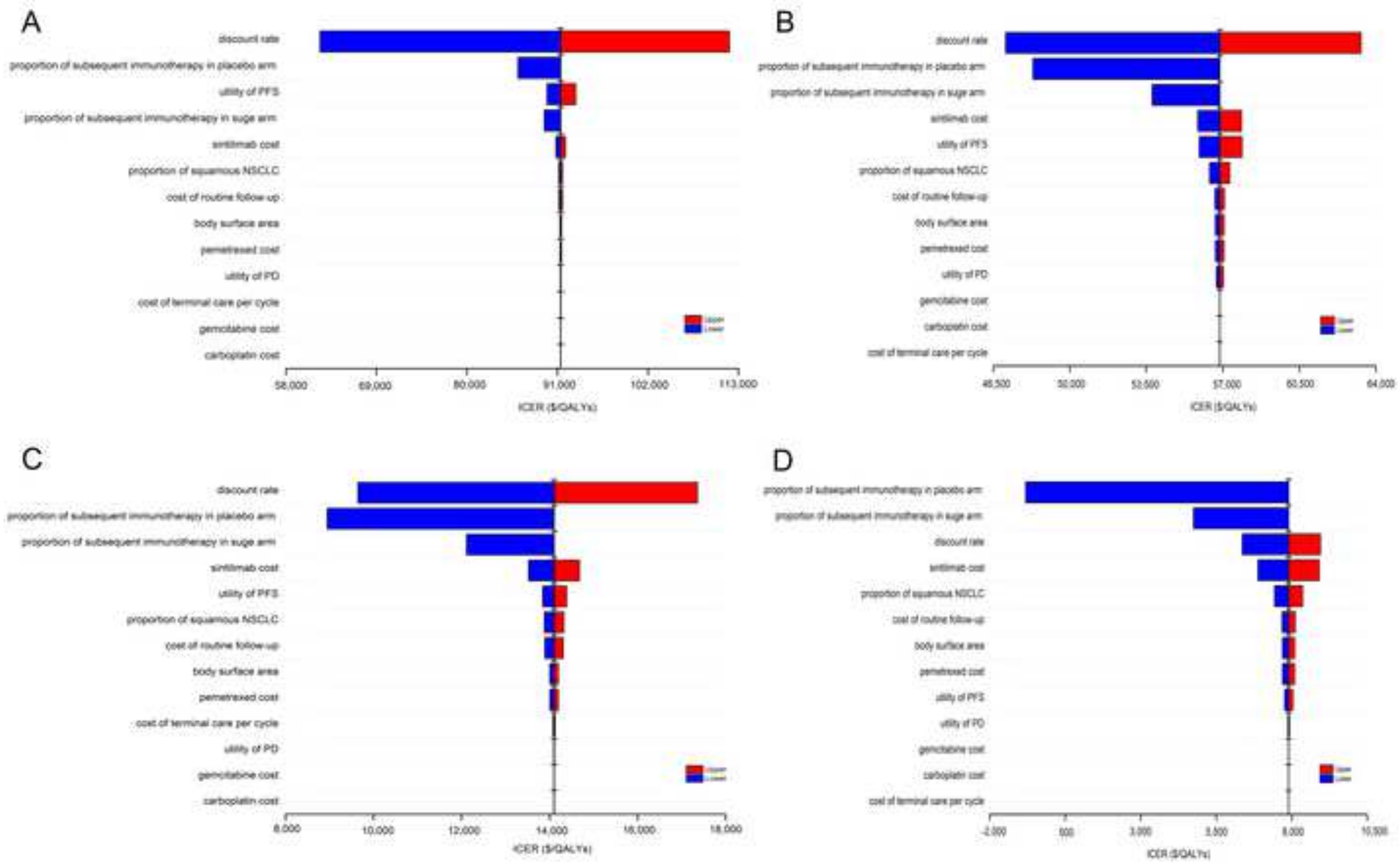

Figure 5

Click here to access/download;Figure;Figure 5.tif

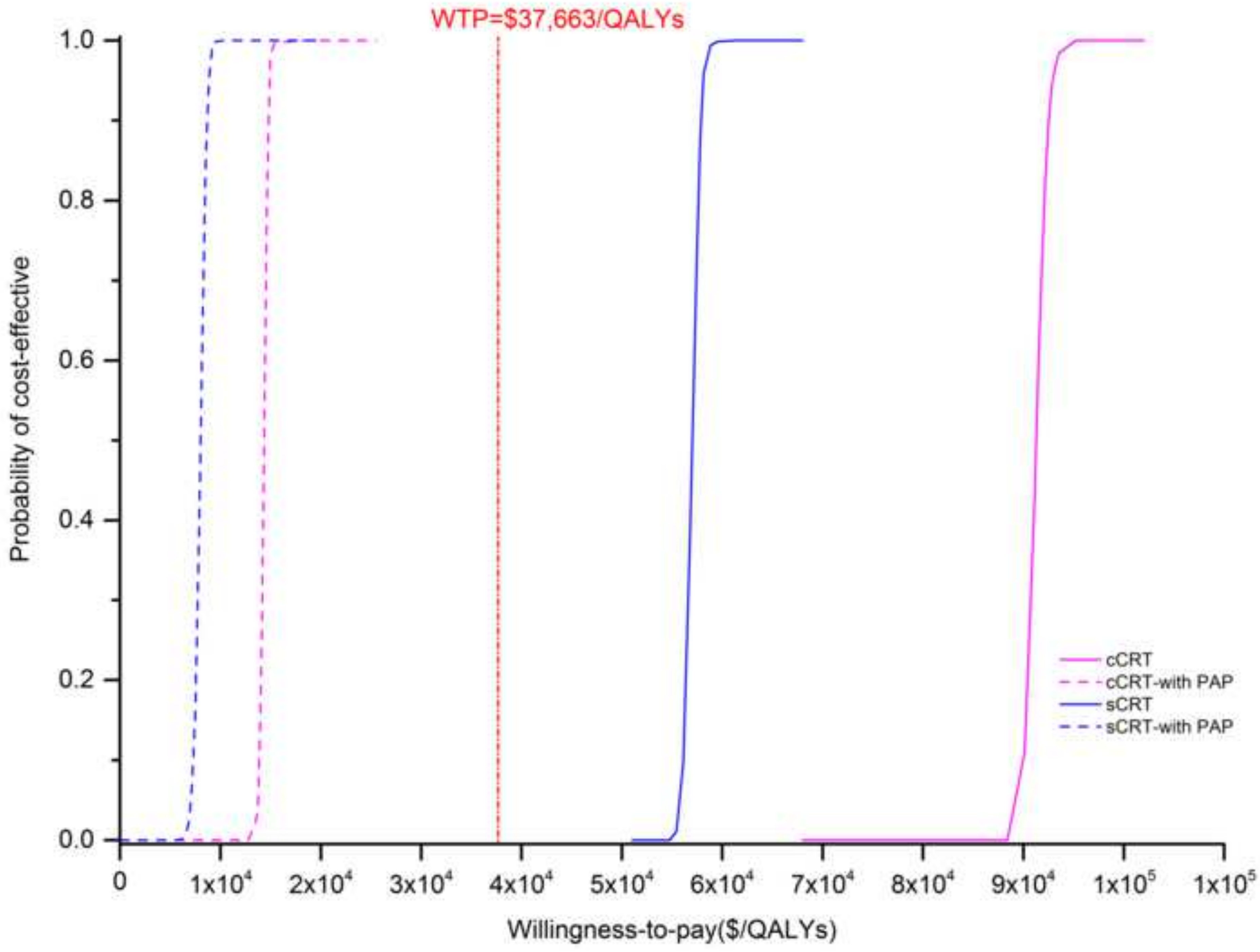

Table S1

Click here to access/download
Supporting Information
Table S1.pdf

Table S2

Click here to access/download
Supporting Information
Table S2.pdf
