# Supplementary material for: A trial-based cost-utility analysis of sugemalimab vs. placebo as consolidation therapy for unresectable stage III NSCLC in China
Source: PLoS One. 2023 Jun 1;18(6):e0286595. doi: 10.1371/journal.pone.0286595 (PMC10234548; doi:10.1371/journal.pone.0286595)
Supplement: Attachment — Submitted filename: Comments for the authors.docx [file pone.0286595.s006.docx]

Dear authors, here are my comments and concern on the manuscript.

1. This manuscript titled “A trial-based cost-effectiveness analysis of sugemalimab vs. placebo as Consolidation therapy for unresectable stage III NSCLC in China” is a very vital and will add relevant findings in the context of China and beyond.
2. However, the ethical issue is not well addressed, simply stated that N/A. The reviewer does not feel that the manuscript meets the journal (PLOS ONE)’s ethical requirements. PLOS ONE requires that research meets all applicable standards for the ethics of experimentation and research integrity. For example, if standard drug is available there, using placebo may not be an ethical.
3. In the abstract, some components were missed. For example, the background is not stated in this section. In this section, the methods part is not comprehensive and exhaustive. For example, what types of costs (direct medical costs and non-medical costs, indirect cost, and intangible costs) the authors considered for this economic evaluation.
4. The authors stated that they estimated costs and health utility in the methods part. Does it cost effective analysis or cost utility analysis?
5. Moreover, some sentences are too long and not clear, so that they need reconstruction. Hence, the manuscript needs minor revision and clarification on its ethical aspect before publication.
